# Prevalence and pattern of comorbidities in chronic rheumatic and musculoskeletal diseases: the COMORD study

**DOI:** 10.1038/s41598-020-64732-8

**Published:** 2020-05-06

**Authors:** Nelly Ziade, Bernard El Khoury, Marouan Zoghbi, Georges Merheb, Ghada Abi Karam, Kamel Mroue’, Jamil Messaykeh

**Affiliations:** 1 Rheumatology department, Hotel-Dieu de France Hospital, Beirut, Lebanon; 2 Rheumatology department, Faculty of Medicine, Saint-Joseph University, Beirut, Lebanon; 3 Gastro-enterology department, Faculty of Medicine, Saint-Joseph University, Beirut, Lebanon; 4 Family Medicine department, Faculty of Medicine, Saint-Joseph University, Beirut, Lebanon; 5 Notre-Dame des Secours University Hospital, Jbeil, Lebanon; 6 Holy Spirit University Kaslik, Jounieh, Lebanon; 7 Zahra University Hospital, Beirut, Lebanon; 8 Monla Hospital, Tripoli, Lebanon

**Keywords:** Epidemiology, Rheumatic diseases

## Abstract

Increased risk of comorbidities has been reported in Rheumatic and Musculoskeletal Diseases (RMD). We aimed to evaluate the prevalence and pattern of comorbidities in RMD patients nationwide, to identify multimorbidity clusters and to evaluate the gap between recommendations and real screening. Cross-sectional, multicentric nationwide study. Prevalence of comorbidities was calculated according to six EULAR axes. Latent Class Analysis identified multimorbidity clusters. Comorbidities’ screening was compared to international and local recommendations. In 769 patients (307 RA, 213 OA, 63 SLE, 103 axSpA, and 83 pSA), the most frequent comorbidities were cardiovascular risk factors and diseases (CVRFD) (hypertension 36.5%, hypercholesterolemia 30.7%, obesity 22.7%, smoking 22.1%, diabetes 10.4%, myocardial infarction 6.6%), osteoporosis (20.7%) and depression (18.1%). Three clusters of multimorbidity were identified: OA, RA and axSpA. The most optimal screening was found for CVRF (> = 93%) and osteoporosis (53%). For malignancies, mammograms were the most optimally prescribed (56%) followed by pap smears (32%) and colonoscopy (21%). Optimal influenza and pneumococcus vaccination were found in 22% and 17%, respectively. Comorbidities were prevalent in RMD and followed specific multimorbidity patterns. Optimal screening was adequate for CVRFD but suboptimal for malignant neoplasms, osteoporosis, and vaccination. The current study identified health priorities, serving as a framework for the implementation of future comorbidity management standardized programs, led by the rheumatologist and coordinated by specialized health care professionals.

## Introduction

Rheumatic and musculoskeletal diseases (RMD) are universally prevalent chronic non-communicable diseases (NCD) with a significant contribution to the Global Burden of Diseases^1^. They are strong determinants of pain, disability^2–4^ and years lived with disability (YLDs) worldwide^5^.

Many patients experience the concurrent presence of more than one NCD, which is a phenomenon known as multimorbidity^6^. NCD may aggregate due to chance -depending on their prevalence in the population-, or due to shared pathophysiologic mechanisms^7,8^. They are likely to act synergistically^9^, causing an overall burden that is larger than the sum of their individual impacts.

In the general population^6^, four distinct patterns of multimorbidity from chronic NCDs were found: low disease probability, cardio-metabolic conditions, respiratory conditions and RMD and depression pattern, with RMD being highly prevalent across all these patterns. All multimorbidity patterns have a direct association with age and are strongly associated with adverse health outcomes such as long-term disability, frequent healthcare utilization, worsened functional status, poorer quality of life^10^ and higher mortality^11,12^.

From the rheumatologists’ perspective, NCD and conditions associated with the RMD are viewed as “comorbidities”. Most rheumatologists consider that it is their responsibility to assess these comorbidities, for several reasons^13,14^. First, some of these comorbidities are more frequently observed in patients with RMD in comparison to the general population. This is clearly the case for cardiovascular diseases^7,15–17^, infections^18,19^ and osteoporosis. This higher prevalence is usually explained by either the activity of the disease itself, by its treatment, or because of an increased prevalence of risk factors such as smoking, hypertension and hyperlipidemia. Second, patients with RMD may receive sub-optimal medical prevention services compared to the general population,^20^,possibly due to the special focus on their rheumatic diseases^21^. In fact, a gap between the screening recommendations and the real practice has been shown in patients with rheumatoid arthritis (RA)^22,23^. Third, some comorbidities might limit therapeutic options thus impacting treatment strategies and jeopardizing the achievement of optimal treatment outcomes^15,24–28^. Finally, there is new evidence suggesting that, although management of Chronic Inflammatory Rheumatic Diseases (CIRDs) improved dramatically over the past decades, comorbidities may have increased^29^.

Although RMD may be heterogeneous, they all seem to share the same healthcare resource utilization, with comorbidities accounting for a substantial proportion of the health costs across all RMD^20,30^.

In RA cohorts^12,23,27,31^, hypertension was found in 31–47%, hypercholesterolemia in 30–32%, diabetes in 10–14% and smoking in 23%. The most frequent associated diseases were osteoporosis (8–24%), depression (12–28%), asthma (1–17%), cardiovascular events (6%), solid malignancies (2–6%) and chronic obstructive pulmonary disease (1–7%). In the COMORA study of 3920 RA, systematic evaluation of comorbidities detected elevated blood pressure in 18%, hyperglycemia in 3.7% and hyperlipidemia in 11% of previously undiagnosed patients. Interestingly, high intercountry variability was observed for both the prevalence of comorbidities and the proportion of subjects complying with recommendations for comorbidities’ screening^23^. Moreover, comorbidities influence the effect of TNFi therapy and are negatively correlated with drug survival^32–34^.

In spondyloarthritis (SpA), according to the international COMOSPA study of 3984 patients, the most frequent risk factors were hypertension (22–34%), smoking (29%) and hypercholesterolemia (27%)^35^. The most frequent comorbidities were osteoporosis (13%) and gastroduodenal ulcer (11%). Again, substantial intercountry variability was observed for comorbidities screening. In psoriatic arthritis (PsA) and psoriasis International Psoriasis and Arthritis Research Team (IPART) cohort of 2254 patients, comorbidity profile rather resembled RA, with 45.1% of hypertension, 49.4% of dyslipidemia, 13.3% diabetes, 75.3% of overweight or obesity, 17.3% smoking. Many risk factors were undertreated (59.2% of hypertension and 65.6% of dyslipidemia)^36^.

To address disparities, the European League Against Rheumatism (EULAR) developed points to consider for reporting, screening for and preventing selected comorbidities in CIRDs, identifying a minimum standardized core set of items to be collected regarding comorbidities in CIRDs in daily practice^13^. In accordance with the EULAR Standardized Operating Procedures, the process comprised recommendations on either the reporting, screening, or prevention of six selected comorbidities domains: ischemic cardiovascular diseases, malignancies, infections, gastrointestinal diseases, osteoporosis, depression. The EULAR taskforce considered that the form would be applicable to all patients with CIRDs, including RA, SpA, connective tissue disorders, crystal-arthropathies as well as polyarticular osteoarthritis.

In Lebanon, the prevalence of RMD in the general population is 14.3% according to the COPCORD study^37^, with a prevalence of 1% for RA and 0.3% of SpA. It has therefore a significant contribution to the global burden of diseases and is increasing with time, particularly with the increasing life expectancy and the development of RMD early diagnosis. There are no specific national estimates for connective tissue disorders and for OA, but rheumatology practices in the country cover the large spectrum of both inflammatory and mechanical diseases. On the other hand, the healthcare system is based, in most cases, on a direct access to the specialist, with poorly developed primary care filtering. Therefore, in most cases, the burden of comorbidities screening lies mostly on the rheumatologist. Finally, no previous study has evaluated the comorbidity pattern in the RMD population in Lebanon and therefore no evidence-based comorbidities’ screening program can be planned.

The primary objective of this study was to evaluate the prevalence and pattern of comorbidities and risk factors in the most frequent RMD in a nationwide study of Lebanese patients. The secondary objectives were to identify multimorbidity patterns and to evaluate the gap between available screening recommendations and routine comorbidities’ screening in daily practice.

## Materials and Methods

### Study design

Observational, cross-sectional, multicentric and nationwide study, from five rheumatology practices across the Lebanese territory, including two university centers and three peripheral clinics.

### Patient recruitment

The rheumatologists from central Beirut, Northern and Southern parts of the country were invited to participate in the study to ensure representativeness. Consecutive adult patients, more than 18 years old, with rheumatoid arthritis (RA), systemic lupus erythematosus (SLE), axial spondyloarthritis (axSpA), peripheral spondyloarthritis (pSpA) or osteoarthritis (OA) “*as diagnosed by the rheumatologist*” were included. Patients were excluded if the current visit was their first visit to the rheumatologist of if they had an association of several CIRDs.

### Data collection

The case report form (CRF) recommended by the EULAR task force^13^ was used to collect the data. Demographics and disease characteristics were collected from the medical file: age, gender, body mass index (BMI), smoking status, alcohol consumption, marital status, profession, social coverage, disease duration, follow-up duration at the rheumatologist’s clinic, current treatment (symptomatic and Disease Modifying Anti-Rheumatic Drugs (DMARDs)), disease activity (DAS-28 for RA, Bath Ankylosing Spondylitis Activity Index (BASDAI) and Ankylosing Spondylitis Disease Activity Score (ASDAS-CRP) for SpA). Additional data was collected by direct patient interview, particularly for vaccination history, Fracture Risk Assessment Tool (FRAX®) calculation and depression screening.

### Comorbidities and risk factors

- Cardiovascular: ischemic cardiovascular diseases, stroke, aneurysm, cardiac dysrhythmia, heart failure, thrombophlebitis; latest hypertension, diabetes, hyperlipidemia and renal function (serum creatinine) screening dates and results; cardiovascular risk score calculation (retrieved when present in the medical file); treatment with anti-platelets, anti-hypertensive, anti-diabetic and hypolipemic drugs.
- Malignancy history (according to clinical reporting, by type), latest screening date by mammography, Pap smear, colonoscopy, Guaiac test, Prostate Specific Antigen (PSA), dermatology visit.
- Infections: history of tuberculosis (active, latent, PPD, interferon gamma test), bacterial, viral (in particular hepatitis B and C, HIV), parasitic and fungal infection; latest vaccination status for influenza, pneumococcus, herpes zoster and HPV as well as up-to-date vaccination for poliomyelitis, diphtheria, tetanus and hepatitis B.
- Gastrointestinal diseases: gastro-duodenal ulcer, Helicobacter pylori infection, previous gastroscopy.
- Osteoporosis: low bone mineral density (BMD) (T-score = <−2.5 DS), osteoporotic fracture and location, anti-osteoporotic treatment, calcium and vitamin D supplementation and FRAX-score. A FRAX risk of  > = 10% was considered as high as per the pharmacological treatment threshold recommended by the Lebanese osteoporosis societies.
- Depression: diagnosed depression, anti-depressant therapy and screening for depression. Screening was done during the medical interview using the Patient Health Assessment Questionnaire (PHQ4).

### Screening of comorbidities

For each patient and each comorbidity/risk factor, optimal screening was calculated and documented as a score of yes (1) or no (0). Optimal screening for each comorbidity/ risk factor was the sum of the cases with a score of 1 divided by the total eligible patients for this screening. Identification of optimal screening according to local (when available) or international recommendations is listed in Table 1 ^16,38–52^.

**Table 1:** Definition of the optimal screening of comorbidities and risk factors.

Comorbidity/Risk Factor	Eligible patients	Optimal Screening	Source
Hypertension	Older than 18 years All RA, axSpA and pSpA patients	Blood pressure yearly	USPSTF^38^ EULAR^16^
Diabetes	Older than 40 years All RA, axSpA and pSpA patients	Free Blood Sugar every 3 years	USPSTF^45^ EULAR^16^
Dyslipidemia	Men older than 35 years Women older than 45 years All RA, axSpA and pSpA patients	Lipid profile every 5 years	USPSTF^46^ EULAR^16^
Breast cancer	Women older than 40 years	Mammography, yearly	Lebanese Recommendations^47^
Cervix cancer	All women	Cervical smear test, every 3 years	USPSTF^48^
Prostate Cancer	Nobody	Recommend against	USPSTF^49^
Colon Cancer	50–75 yo	Colonoscopy every 5 to 10 years Or Gaiac every 2 years	USPSTF^50^
Skin Cancer	No clear recommendation	Dermatology visit, at least once	USPSTF^51^
Influenza Vaccination	Older than 18 years	Yearly	CDC^52^
Pneumococcus Vaccination	All patients older than 65 years RA and SpA patients taking biologic DMARDs	Every 5 years	CDC^39^ EULAR^18^
HPV Vaccination	Women 18–26 years, Men 18–21 years		CDC^40^
Herpes Zoster Vaccination	Older than 60 years	Not available in Lebanon	CDC^41^
Osteoporosis	Older than 65 yo or Fragility fracture All RA patients	DXA, at least once	Lebanese Recommendations (OSTEOS)^42^
DTP vaccination	All patients	Ever	CDC^43^
Depression	All adults	Clinical screening	USPSTF^44^

CDC: Center for Disease Control. EULAR: European League Against Rheumatism. USPSTF: U.S. Preventive Services Task Force.

The CRF was completed by a medical intern and a study nurse using a review of the medical record and an interview at the study visit. The data was entered in a Microsoft Excel database.

### Statistical analysis

A descriptive analysis of all patients was performed and a comparison between the five rheumatologists’ data profile was done. The prevalence (and 95% CI) of each comorbidity and risk factor were estimated (Wald method). The number of comorbidities was correlated with predictive factors using Poisson Regression. Latent Class Analysis (LCA) was used to identify clusters of multimorbidity, which included the RMD and the most frequent comorbidities in the model. It consists of a measurement model in which individuals can be classified into mutually exclusive and exhaustive types, or latent classes, based on their pattern of categorical variables. The percentage of optimally screened patients according to the recommendations was calculated. Optimal screening (binary) was correlated with predictive factors using binary logistic regression. Analyses were conducted using the statistical software IBM SPSS Statistics 25 and XLSTAT 18.07 (LCA analysis).

### Sample size calculation

The sample size was based on the width of the 95% CI of the proportion of expected events (the prevalence of comorbidities), assuming that a 753-patient sample would allow an observed 2% prevalence of a comorbidity to be estimated with a precision of 1% (95% CI 1% to 3%). (http://sampsize.sourceforge.net/iface/).

### Ethical considerations

The study was approved by the ethical committee of the Saint-Joseph University (Approval number TFEM2016/48), Beirut, which acts in accordance with the ethical standards laid down in the 1964 Declaration of Helsinki and its later amendments. All physicians gave their written informed consent to interview the patients and to access the data on file and to interview the patients, all patients gave their oral informed consent to answer the study questionnaire.

## Results

### Population characteristics

We recruited 769 patients between 2016 and 2018 (307 RA, 213 OA, 63 SLE, 103 axSpA, 83 pSpA). Significant differences in patients’ demographics were found across the diseases (Table 2). Mean age was 55.8 years (SD 13.9), (lowest in SLE 42.8 yo, highest in OA 63.6 yo, p < 0.001). Female gender was 76.9% (lowest in axSpA 43.7%, highest in SLE 93.7%, p < 0.001). 42.9% were professionally active (lowest in OA 26.1%, highest in axSpA 79.5%, p < 0.001, probably reflecting the age and gender differences). Most of the patients had a partial or total social coverage (77.1%), the rest being covered by the ministry of health (p = 0.595). Disease duration ranged from 84.5 months in OA to 103.5 months in RA (p = 0.013). 29.9% were previous or current smokers, 22.1% were current smokers (11.1% in SLE to 28.2% in axSpA, p < 0.001) and 2.9% drank alcohol regularly (1.6% in RA to 8.2% in axSpA, p = 0.562). Mean BMI was in the overweight range 27.02 kg/m^2^ (SD 4.71). There was no difference in the disease distribution between the five rheumatology clinics for the demographic and disease characteristics (p = 0.301).

**Table 2:** Demographic and disease characteristics of the 769 patients.

	RA	OA	SLE	axSPA	pSpA	p
N (%)	307 (39.9)	213 (27.7)	63 (8.2)	103 (13.4)	83 (10.8)	
Age: years (SD)	57.3 (12.8)	63.6 (10.7)	42.8 (13.7)	46.7 (11.6)	51.2 (12.9)	<0.001
Female: %	82.1	81.2	93.7	43.7	74.7	<0.001
Smoking: %	28.0	18.3	11.1	28.2	10.80	<0.001
Alcohol: %	1.6	4.8	3.8	8.2	2.2	0.562
BMI kg/m^2^ (SD)	27.1	27.0	25.6	26.8	27.9	0.065
Profession: active (%)	39.5	26.1	48.7	79.5	53.1	<0.001
Disease duration, months	103.5	84.5	93.5	94.2	82.6	0.013
Conventional DMARDs: %	70.4	n/a	72.2	54.2	72.2	0.196
Biologic DMARDs: %	30.1	n/a	n/a	46.8	33.3	0.105
Corticosteroids: %	21.0	2.5	38.9	4.1	15.0	<0.001
NSAIDs (chronic): %	27.2	2.60	24.1	41.1	26.3	<0.001

RA = Rheumatoid Arthritis. OA = Osteoarthritis. SLE = Systemic Lupus Erythematosus, axSpA = Axial Spondyloarthritis. pSpA= Peripheral Spondyloarthritis. BMI: Body Mass Index. DMARDs: Disease-Modifying Anti-Rheumatic Drugs. NSAIDs: Non-Steroidal Anti-Inflammatory Drugs. DAS-28: Disease Activity Score. ESR: Erythrocyte Sedimentation Rate. n/a = not applicable.

### Prevalence of comorbidities

The most common comorbidities were cardiovascular risk factors (hypertension 36.5%, hypercholesterolemia 30.7%, obesity 22.7%, smoking 22.1% and diabetes 10.4%) and cardiovascular diseases (myocardial infarction/angina 6.6%, dysrhythmias 4.6% and stroke 2.1%) (CVRFD) Table 3.

**Table 3:** Prevalence of most frequent comorbidities and risk factors, by disease (all values are %).

	Comorbidity/Risk Factor	RA	OA	SLE	axSpA	pSpA	Total	95% CI	p
Cardiovascular Diseases	**MI/ Angina**	5.9	11.3	1.6	3.9	4.8	6.6	4.8–7.7	**0.018**
	Cardiac failure	2.3	0.0	0.0	0.0	0.0	0.9	0.1–2.4	**0.031**
	Thrombophlebitis	1.6	2.3	4.8	1.9	2.4	2.2	0.7–3.3	0.654
	Dysrhythmia	5.5	3.8	7.9	1.9	3.6	4.6	3.0–6.5	0.106
	Stroke	0.7	5.2	1.6	1.0	1.2	2.1	0.7–3.29	**0.007**
	Aneurysm	1.6	0.5	0.0	0.0	0.0	0.8	0.1–1.7	0.279
Cardiovascular Risk Factors	**Hypertension**	36.2	48.8	25.4	17.5	38.6	36.5	32.7–41.1	**<0.001**
	**Hypercholesterolemia**	30.3	42.3	19.0	17.5	28.9	30.7	26.4–34.5	**0.001**
	**Diabetes**	9.8	14.6	7.9	5.8	9.6	10.4	6.9–13.1	0.144
	**Obesity (BMI** > **30 kg/m**^**2**^**)**	26.2	21.6	12.7	20.8	23.3	22.7	19.–26.0	0.202
	**Smoking**	28.0	18.3	11.1	28.2	10.8	22.1	18.9–26.9	**<0.001**
Malignant Neoplasms	All malignancies	5.9	4.3	2.2	3.7	3.6	4.2	4.3–8.2	0.244
	Breast cancer	3.2	1.6	0.0	2.4	4.3	2.4	1.3–3.4	0.581
	Prostate cancer	1.1	1.6	1.9	1.2	0.0	1.2	0.09–1.9	0.921
	Colon cancer	1.1	0.6	0.0	1.2	0.0	0.6	0.07–1.2	0.633
Infections	Tuberculosis*	2.7	1.6	0.2	1.2	2.2	1.8	0.6–2.9	0.598
	Bacterial	12.2	12.5	8.8	5.5	0.0	10	7.5–12.7	0.790
	Other**	4.4	4.8	0.7	4.9	2.2	3.2	2.2–5.6	0.792
Osteoporosis	**Osteoporotic Fractures**	11.4	17.4	1.6	4.9	6.0	10.8	7.3–13.3	**<0.001**
	**FRAX** > 10%***	25.1	24.4	22.2	6.8	10.8	20.7	17.5–22.7	**<0.001**
Gastro-Intestinal	**Gastric Ulcer**	5.5	7.1	5.8	2.4	6.5	5.5	3.4–7.9	0.695
	Helicobacter pylori	1.6	3.2	1.9	0.0	0.0	1.8	0.6–3.3	0.671
	IBD	0.5	0.6	0.0	23.3	7.5	4.3	2.5–6.0	**<0.001**
Psycho- logical	**Depression (screened)**	22.1	15.0	27.0	7.8	16.9	18.1	15.0–21.7	**0.004**
	**Depression (treated)**	18.6	11.7	19.0	6.8	12.0	14.4	11.0–17.45	**0.019**

*Tuberculosis: active and latent.

**Other infections: Viral, parasitic and fungal (Hepatitis, CMV, Herpes, Isospora, Toxoplasma, Candida).

***FRAX was available in 380 patients (49.4%).

Comorbidities and risk factors with prevalence >5% are in bold. p-values < 0.05 are in bold.

They were followed by osteoporosis, with 20.7% of patients with high FRAX risk for major fractures (highest in RA 25.1%, lowest in axSpA 6.8%, p < 0.001), and depression 18.1% (highest in SLE (27.0%) and lowest in axSpA (7.8%), p = 0.004). Osteoporotic fractures were found in 9.3% of patients (highest in RA 25.1% and lowest in SLE 6.8%, p < 0.001). The main site of fracture was vertebral (6.1%), followed by wrist (3.2%) and hip fracture (2.6%). 74.4% were taking vitamin D supplementation, 49.4% were taking calcium supplementation, 13.2% were taking anti-osteoporotic drugs (bisphosphonates 9.9%, denosumab 1.7%, raloxifene 0.6%, strontium ranelate 0.6% and teriparatide 0.4%).

A history of malignancies was found in 4.2% of patients, with the highest prevalence for breast (2.4%), prostate (1.2%) and colon (0.6%) cancers. Hematologic cancers were found in 0.4% of patients.

Tuberculosis history (latent and active) was found in 1.8% of patients. Recent history of bacterial infections (Salmonella, Pneumococcus, Brucella, E coli, Klebsiella, Pseudomonas) was found in 10% of patients. History of viral, parasitic and fungal infections (Hepatitis, CMV, Herpes, Isospora, Toxoplasma, Candida) was found in 3.2% of patients.

Gastric ulcer history was found in 5.5% of patients, Helicobacter pylori infection in 1.8% and Inflammatory Bowel Diseases (IBD) in 4.3%, highest in axial (23.3%) and peripheral (7.5%) SpA as it would be expected (p < 0.001).

The total number of comorbidities per patient was highest for OA (1.8) and lowest for axSpA (0.8), p < 0.001. In multivariate analysis, age (p < 0.001), higher BMI (p < 0.001) and biologic therapies (p = 0.05) were significantly associated with the number of comorbidities.

### Multimorbidity patterns

**Figure 1:**
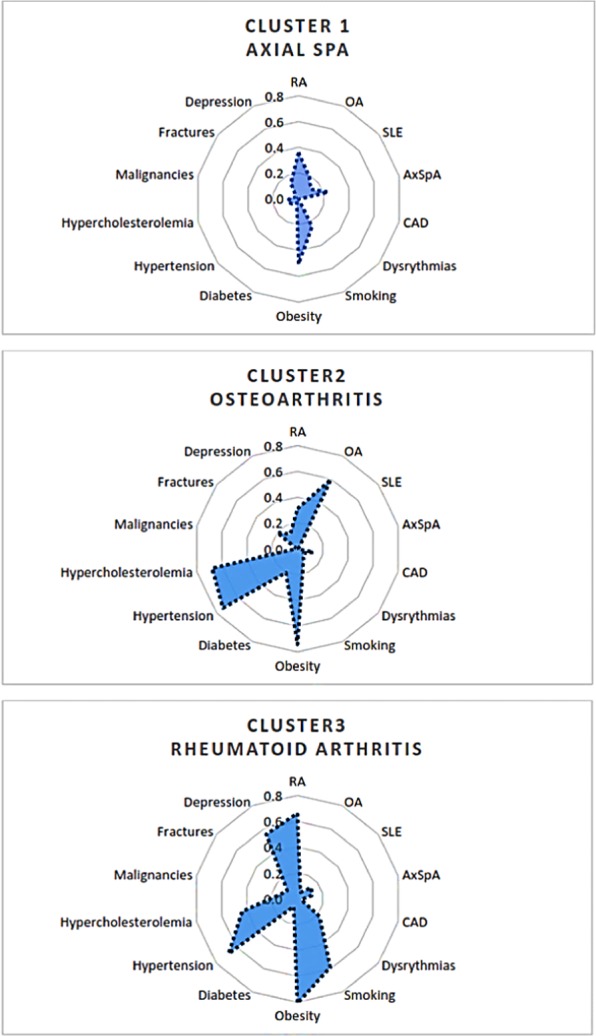
Multimorbidity patterns identified by Latent Class Analysis: Osteoarthritis, Rheumatoid Arthritis and axial Spondyloarthritis patterns. AxSpA: Axial SpondyloArthritis, OA: Osteoarthritis, CAD: Coronary Artery Disease, RA: Rheumatoid Arthritis. Each comorbidity is represented by its prevalence in each spidergram (for example, Smoking’s prevalence is 60% in Cluster 3).

LCA analysis identified 3 main clusters of multimorbidity: OA, RA, axSPA (Fig. 1).

RA pattern had the highest clusters of comorbidities: obesity, smoking, coronary artery diseases, hypertension, hypercholesterolemia, osteoporotic fractures and depression. OA pattern comprised obesity, hypertension, hypercholesterolemia, fractures and malignancies. axSpA pattern had the lowest disease clustering.

### Optimal screening for comorbidities and risk factors

The most optimal screening was found for cardiovascular risk factors (98% for hypertension, 96% for diabetes and 93% for hypercholesterolemia). However, cardiovascular risk score was not calculated in the medical files Table 4.

**Table 4:** Optimal screening for comorbidities and risk factors, by disease (all values are %).

	Comorbidity/Risk Factor	RA	OA	SLE	axSpA	pSpA	Total
Cardio-vascular	Hypertension	96	100	93	96	98	98
	Hypercholesterolemia	95	97	82	98	88	93
	Diabetes	98	99	87	99	93	96
Malignant Neoplasms	Mammogram	44	76	40	46	30	56
	Pap smear	34	34	21	24	33	32
	Colonoscopy	16	16	50	48	29	21
Vacci-nation	Influenza	30	10	16	30	20	22
	Pneumococcus	24	3	17	23	8	17
Osteoporosis	Vitamin D supplementation	79	83	71	49	70	74
	DXA	47	72	22	83	67	53
Depression	Depression screening	1	0	0	3	5	1

DXA was prescribed in 53% of correct indications, FRAX was available in 49% of patients, 74% of patients were supplemented with vitamin D.

As for malignancies, mammograms were the most optimally prescribed (56%) followed by pap smears (32%) and colonoscopy (21%).

Correct vaccination (influenza and pneumococcal) was found only in 22% and 17% respectively. Childhood vaccinations were vaguely remembered and were not included because of high recall bias and the absence of a vaccination record. No vaccinations were found for HPV and Herpes zoster virus.

Depression was very poorly screened according to the patient’s file (1%).

Optimal screening was associated with the patient’s age and the physician’s university setting for all comorbidities and risk factors (p < 0.001). Moreover, it was associated with disease duration (p 0.017) and social coverage (p 0.036) for gynecologic cancer screening, with female gender for DXA prescription (p < 0.001) and with biological treatment (p < 0.001) and corticosteroid therapy (p 0.008) for adequate influenza and pneumococcus vaccination.

## Discussion

This is the first prevalence study assessing comorbidities and risk factors and their screening in a nationwide population of RMD (CIRD and osteoarthritis simultaneously) in Lebanon.

### Prevalence of comorbidities

In our study, the main risk factors and comorbidities followed three main prevalence axes: cardiovascular, osteoporosis and depression.

Cardiovascular risk factors were the most prevalent with 36.5% of hypertension, 30.7% of hypercholesterolemia, 22.7% of obesity, 22.1% of smoking, and 10.4% of diabetes. These risk factors were statistically different across diseases, with the highest prevalence in the osteoarthritis arm, due to the age difference most evidently. Our numbers were consistent with the WHO and Lebanese Ministry of Health (MOH) reports for hypertension (28.8% to 41.3%), hyperlipidemia (32%), but slightly lower for smoking (32% (WHO) to 38.8% (MOH)), diabetes (18%) and obesity (27.4%) than the general Lebanese population aged 50 years and above^2,53,54^. We had a similar rate of myocardial infarction and stroke^53,55^.

Osteoporosis was the second most prevalent comorbidity axe, with a prevalence of 20.7% as identified by a FRAX score >10% and of 10.8% as identified by osteoporotic fractures. This number is slightly higher than the reported 13% osteoporotic bone mineral density at the femoral neck reported in the Lebanese patients aged 50–79 yo^56^.

The third axe of comorbidity was depression, with 18.1% of patients detected by the questionnaire, slightly more than the patients treated with anti-depressant drugs (14.4%). Depression was higher in SLE, and lowest in axSpA. It seemed to be higher than the previously reported 9.9% prevalence in a Lebanese cross-sectional study^57^ and was associated with female gender as in the Portugese National Health multimorbidity Survey^6^.

Malignant neoplasms were identified in around 4% of patients and seemed to be lower than in the general Lebanese population, although direct comparison cannot be made. However, malignant neoplasms site proportions seemed to be respected^58^.

Compared to the COMORA study^23^, our RA patients had the same age (56 ± 13 yo in COMORA vs 57.3 ± 12.8 yo in COMORD), and same gender balance (81.7% female vs 76.9%). Disease duration (9.6 y vs 8.3 y) and biological agents use (39% vs 30.1%) were slightly lower. We found almost similar prevalence of hypertension (40% vs 36.2%), hypercholesterolemia (31.7% vs 30.3%) and colon cancer (1%), less diabetes (14% vs 9.8%), stroke (2% vs 0.5%), prostatic cancer (2% vs 1%), breast cancer (2% vs 0.7%) and gastro-intestinal ulcers (11% vs 5.5%). On the other hand, we had more smokers in our RA population (20% vs 28%) and higher depression prevalence (15% vs 22.1%).

Compared to the COMOSPA study, our axSpA population had similar age (44 yo vs 47 yo), less males (65% vs 56.3%), shorter disease duration (8.2 vs 7.85 years) but slightly higher biological therapy (43.9 vs 46.8%). We found lower diabetes prevalence (8.8% vs 5.8%), less hypertension (33.5% vs 17.5%), hypercholesterolemia (27.3% vs 17.5%), gastro-intestinal ulcers (10.7% vs 6.5%) and osteoporosis (13.4% vs 6.8%). Malignant neoplasms prevalence were too low to be compared. Moreover, compared to the recently published French study by Claudepierre *et al*., which includes a large database of severe forms of SpA, mean age 50.4 yo, our study found a similar prevalence of diabetes (6.2% vs 5.8–9.6%), of malignancies (3.9% vs 3.7–3.6%) and a higher prevalence of hypertension (4.7% vs 17.5%), ischemic heart disease (2.2% vs 3.9–4.8%), arrythmias (1.7% vs 1.9–3.6%), stroke (0.8% vs 1–1.2%), IBD (3.5% vs 7.5–23.3%) and depression (3.1% vs 7.8–16.9%) although these diseases may be underestimated in the French study due to the inclusion of diseases registered as long-term fully covered only (“Affections de Longue Durée”)^59^.

High percentage of smokers in our COMORD population could reflect the high smoking prevalence found in the Lebanese general population. On the other hand, higher depression rates could be attributed to the legacy of several brutal wars in Lebanon which has led to chronic psychological distress. These two preventable conditions should be considered seriously, as rheumatologists should actively promote smoking cessation and depression screening, and ideally plan automatic referral pathways when these conditions are detected.

Fibromyalgia was not included in the EULAR form. Although it’s prevalent in RMDs and is usually recognized by the rheumatologist, it is often not noted in the medical file and is difficult to diagnose with confidence by the medical intern and the study nurse who performed the interview. Thus, due to the difficulty of screening of such a complex disease, we preferred not to add it in our form.

Multimorbidity patterns with RA resembles the fourth pattern identified in the Portuguese National Health Survey^6^, with an clustering of RMD, osteoporosis, depression and cardiovascular risk factors (such as hypertension and diabetes), suggesting a potential synergistic negative effect on outcomes.

The higher comorbidities prevalence and multimorbidity burden found in OA compared to the other diseases, particularly axSpA, can be explained by the large age differences between the two groups. In fact, the mean age was 63.6 years in OA, whereas it was 46.7 in axSpA, which is probably the main factor driving the difference in multimorbidity.

### Optimal screening of comorbidities

Optimal screening was highest for CVRF (>93%) but was largely sub-optimal for osteoporosis (FRAX available in 49%, 53% of correctly prescribed DXA, although 74% were supplemented with vitamin D), malignant neoplasms screening (less than 50%), vaccination (22% for influenza and 17% for pneumococcus), and almost absent for depression (1%).

Compared to the COMORA study, we had a higher screening in our population for cardiovascular risk (59% vs >93%), higher influenza (25% vs 30%) and pneumococcal vaccination (17% vs 23%) and lower screening for malignant neoplasms (51% vs 44% for breast cancer, 59% vs 34% for uterine cancer, 27% vs 15% for colon cancer) and for osteoporosis (58% vs 47%).

Compared to the COMOSPA study, we also had also a higher cardiovascular screening (50.5% vs >93%), higher colon cancer screening (32.7% vs 50%, probably biased by the fact that the axSpA patients are easily prescribed colonoscopies as screening for inflammatory bowel disease associated extra-articular manifestation), slightly higher breast cancer screening (44% vs 45%) and pneumococcal vaccination (17.4% vs 22%). We had lower uterine cancer screening (39.8% vs 20%) and influenza vaccination (38% vs 30%).

The incomplete vaccination rates are almost universally found, as well as suboptimal malignant neoplasms screening^23,35,60^. In this case, the contribution of rheumatology nurses may improve the vaccination rates and comorbidities management, as it was suggested in the nurse-led program of the COMEDRA study^61^.

Several types of bias are inherent in our study. The prevalence of some comorbidities may be overestimated because of diagnostic bias, in that patients with RMD closely followed at a rheumatology practice may be offered more screening for comorbidities known to be associated with their RMD. On the other hand, the prevalence of some other comorbidities may be underestimated because of selection bias, as patients with life-threatening conditions as malignant neoplasms or severe myocardial infarction or stroke, may have been unable to participate in the study. Another bias is the lack of direct comparison general population arm. This comparison arm however is difficult to find, since patients recruited at primary care clinics may be offered higher screening than the general population, just for the reason of being medically followed. Finally, we didn’t use common validated comorbidity indices, because they are usually validated only in one type of RMD, mostly RA^62,63^. Instead, we preferred using the EULAR points to consider as they cover all RMD simultaneously.

A main limitation of our study is the small sample size, which reflects the small general population size (around 4 million inhabitants) and the frequent management of RMDs by non-rheumatology specialists, i.e. orthopedic surgeons, internists, family physicians…

Despite these limitations, this is the first study in the region that gives an estimate of the prevalence of major comorbidities associated with RMD, using a EULAR-recommended questionnaire, and that identifies the gaps between recommended screening and actual practice. Including an OA arm in our study is considered as an added value as it reflects the local large spectrum rheumatology practice covering both mechanical and inflammatory RMDs. It can be viewed as a control arm for inflammatory RMDs,. Moreover, the inclusion of the polyarticular form is recommended by the EULAR comorbidity taskforce. We had no differences between the different Lebanese practices, and the estimated prevalence were relatively consistent with the figures published internationally, which gives the study good external validity, despite the low sample size. Since the file review was complemented by a face-to-face interview, there was no missing data, except for childhood vaccination which was vaguely remembered and impossible to retrieve due to the absence of childhood vaccination records.

## Conclusion

Comorbidities have a high prevalence in RMD and should be considered in the patient’s routine work-up and integrated in a holistic approach to the patient. Although cardiovascular screening seems satisfactory, major efforts should be made to promote smoking cessation, improve osteoporosis, malignant neoplasms and depression screening and implement adequate vaccination. The current study identified health priorities, serving as a framework for the implementation of future comorbidity management standardized programs, led by the rheumatologist and coordinated by specialized health care professionals.
